# A case matched study examining the reliability of using ImPACT to assess effects of multiple concussions

**DOI:** 10.1186/s40359-017-0184-1

**Published:** 2017-04-28

**Authors:** Trevor Barker, Stephen A. Russo, Gaytri Barker, Mark A. Rice, Mary G. Jeffrey, Gordon Broderick, Travis J. A. Craddock

**Affiliations:** 1 Department of Psychology & Neuroscience, Nova Southeastern University, Ft. Lauderdale, 33314 FL USA; 2 Department of Neurology, Thomas Jefferson University, Philadelphia, 19107 PA USA; 3 Department of Psychiatry and Human Behavior, Thomas Jefferson University, Philadelphia, 19107 PA USA; 4 Department of Clinical Immunology, Nova Southeastern University, Ft. Lauderdale, 33314 FL USA; 5 Institute for Neuro-Immune Medicine, Nova Southeastern University, Ft. Lauderdale, FL 33314 USA; 6 Department of Computer Science, Nova Southeastern University, Ft. Lauderdale, 33314 FL USA

**Keywords:** Mild traumatic brain injury, Neurocognitive testing, ImPACT, Sex differences, Concussion history

## Abstract

**Background:**

Approximately 3.8 million sport and recreational concussions occur per year, creating a need for accurate diagnosis and management of concussions. Researchers and clinicians are exploring the potential dose-response cumulative effects of concussive injuries using computerized neuropsychological exams, however, results have been mixed and/or contradictory. This study starts with a large adolescent population and applies strict inclusion criteria to examine how previous mild traumatic brain injuries affect symptom reports and neurocognitive performance on the Immediate Post-concussion Assessment and Cognitive Testing (ImPACT) computerized tool.

**Methods:**

After applying exclusion criteria and case matching, 204 male and 99 female participants remained. These participants were grouped according to sex and the number of previous self-reported concussions and examined for overall differences on symptoms reported and scores obtained on the ImPACT neurocognitive battery composites. In an effort to further reduce confounding factors due to the varying group sizes, participants were then case matched on age, sex, and body mass index and analyzed for differences on symptoms reported and scores obtained on the ImPACT neurocognitive battery composites.

**Results:**

Case matched analysis demonstrated males with concussions experience significantly higher rates of dizziness (*p* = .027, η^2^ = .035), fogginess (*p* = .038, η^2^ = .032), memory problems (*p* = .003, η^2^ = .055), and concentration problems (*p* = .009, η^2^ = .046) than males with no reported previous concussions. No significant effects were found for females, although females reporting two concussions demonstrated a slight trend for experiencing higher numbers of symptoms than females reporting no previous concussions.

**Conclusions:**

The results suggest that male adolescent athletes reporting multiple concussions have lingering concussive symptoms well after the last concussive event; however, these symptoms were found to be conflicting and better explained by complainer versus complacent attitudes in the population examined. Our results conflict with a significant portion of the current literature that uses relatively lenient inclusion and exclusion criteria, providing evidence of the importance of strict inclusion and exclusion criteria and examination of confounding factors when assessing the effects of concussions.

**Electronic supplementary material:**

The online version of this article (doi:10.1186/s40359-017-0184-1) contains supplementary material, which is available to authorized users.

## Background

A traumatic brain injury (TBI) is any damage to the brain resulting from an external force that can potentially lead to serious clinical outcomes. Estimates indicate that at least 1.4 million Americans acquire a TBI annually, marking it as the third highest cause of injury-related deaths in the U.S., [1, 2] especially for children and young adults. These reported injuries cost $60 billion per year [3]; however, this number is considered an underestimate since many that suffer from mild TBIs often fail to seek medical services [1, 2]. Taking unreported injuries into account increases the number of estimated TBIs to 3.8 million annually for sports and recreational activities alone [2], with 75–90% being “mild” in nature [4]. TBI is clearly a public health concern.

From an athletic perspective, mild TBIs (mTBI), also referred to as “concussions,” have recently captured the attention of the media, the sports community, and practitioners in the sports medicine disciplines [5], especially for high-risk sports (e.g., boxing, wrestling, American football, ice hockey, soccer, etc.). While the majority of these mTBI injuries are acute with symptoms resolving within 7–10 days for adults, or longer for children [6], long-term chronic consequences can result from single as well as multiple brain traumas [6]. Thus, the initial diagnosis and management of mTBI is of such great importance that it may help the athlete avoid persistent TBI related conditions, including post-concussion syndrome (PCS), or reduce the likelihood of developing chronic TBI related conditions such as chronic traumatic encephalopathy (CTE) [7].

From a neuro-cognitive perspective, researchers and clinicians are exploring the potential dose-response cumulative effects of concussive injuries. However, results have been mixed with some studies finding no measurable effect on neuropsychological profiles and concussion symptoms [5, 8]; effects only on symptoms [9]; and effects only on neuropsychological measures such as verbal memory [10, 11], visual memory [10], and attention/concentration [12]. Such differences may arise due to insufficient sample sizes, leading to lack of stringent inclusion/exclusion criteria, absence of case matching, and mixing of sexes. For example, Iverson et al. [8] investigates a seemingly large cohort of 867 male participants without case matching, but this population only possesses a maximum of 54 individuals with two previous concussions, limiting the group sizes for potential case matching even before applying any exclusionary criteria. In a comparable group size of 786 male athletes, Iverson et al. [11] did apply case matching, but not exclusions, for participants with 3 or more concussions, resulting in groups of 26. Applying exclusionary criteria is only expected to decrease the group size, and statistical power of the analysis in such studies. However, the application of exclusionary criteria is critical for proper interpretation of the results. Baseline neurocognitive scores in athletes with attention deficit–spectrum disorders and/or learning disability show significantly lower verbal memory, visual memory, and visual motor processing speed scores, along with significantly higher reaction time, and symptom scores [13, 14]. The mixing of sexes is also of particular concern as there is sufficient evidence to suggest males and females respond differently to concussions [10, 15].

In the present study, we attempt to address limitations of current literature by utilizing a large sample database of baseline neuropsychological profiles and post concussive symptom reports as well as applying stringent inclusion and exclusion criteria. In an effort to further reduce confounding factors, we case matched the remaining sample on age, gender, and body mass index (BMI) before examining how multiple self-reported concussions affect neurocognitive performance and reported symptoms.

## Methods

### County-wide clinical management program

The current study is based on archival, de-identified data that was obtained via a community-wide concussion initiative where a university-based sports medicine clinic located within the Southeastern portion of the United States partnered with the local school board and the county athletic association to provide concussion education, evaluation, and management services. Permission to access this database was obtained from the Nova Southeastern University’s Sports Medicine Clinic. As part of this initiative, county school athletes aged 10-19 were administered a baseline neurocognitive screening prior to the start of their sport season. This screening was performed via the Immediate Post-concussion Assessment and Cognitive Testing (ImPACT) v2.1, a widely used neuropsychological testing battery designed for assessing and managing the neurocognitive aspects of sports-related concussion [16]. The ImPACT data used in this study was obtained between 2011 and 2014.

### Participants

Baseline scores for a total of 26,240 (16,375 male and 9865 female) athletes aged 10 to 19 years (*M* = 15.4 years, SD = 1.27) were available for the present study. Athletes were from a wide variety of sports including football, lacrosse, soccer, wrestling, gymnastics, swimming, cheerleading, basketball, baseball, and tennis.

### Inclusion and exclusion criteria

Exclusion criteria for the present study consisted of factors that could adversely affect cognitive performance and/or alter symptom reporting. More specifically, exclusion criteria for the present study were adapted from previous published findings in the area of sport-related concussion and included a self-reported history obtained via the ImPACT detailing treatment for substance abuse [10, 15], psychiatric disorder [10, 15], special education enrollment [15, 17], repeated years of schooling [15, 18], diagnosis of attention-deficit disorder (ADD) and attention-deficit/hyperactivity disorder (ADHD) [10, 19, 20], learning disability [10, 17, 19, 21], autism, speech therapy [15]; different first language than test administered language [21]; and a self-reported history of brain surgery. Individuals that did not complete baseline testing or whose test performance was flagged as being potentially invalid by the ImPACT were also removed before analysis. After applying exclusion criteria, 18,415 (10,879 male and 7536 female) adolescent athletes were included in the analyses.

### Symptom and neurocognitive assessment

ImPACT consists of three sections that provide information to aid in the clinical evaluation of concussion: Demographics Information/Health History Questionnaire; Current Concussion Symptoms/Conditions; and Neuropsychological Functioning. Embedded in the Current Concussion Symptoms/Conditions section is the Post-Concussion Symptom Scale (PCSS) which asks participants to rate their current severity of 22 symptoms associated with concussion on a 7-point Likert scale [22]. The Neuropsychological Functioning section is comprised of six modules that are used to test an individual’s neuropsychological functioning: Word Memory, Design Memory, X’s and O’s, Symbol Matching, Color Match, and Three Letter Memory. The results of these modules are condensed to provide five neuropsychological functioning “composite” scores describing Verbal Memory, Visual Memory, Visual-Motor Speed, Reaction Time, and Impulse Control. Although the reliability of ImPACT scores has been questioned [23, 24], previous studies have shown ImPACT scores to be reliable at 1 month [25], 1 year [26], and 2 years [27]. The validity and reliability of ImPACT in the assessment of sport-related concussion has also been demonstrated previously [18, 28].

### Statistical analysis

Data were analyzed with IBM SPSS for Windows, version 22 [29]. Participants were grouped according to sex and the number of previous self-reported concussions: 0, 1, and 2. The majority of participants reported zero concussions (*n* = 13,329), 613 reported a history of one concussion, and 103 reported a history of two concussions. The remainder of the participants reported between three and ten previous concussions, or failed to provide an answer to this question. Groups with three or more concussions were not considered in this study, as their small size would significantly reduce the statistical power of the analysis. In an effort to reduce confounding factors identified by previous research and variance among groups due to the differing group sizes, participants from each concussion group were then case matched on age [15, 30], sex [10, 15, 31], and BMI [10, 32, 33] utilizing an in-house MATLAB [34] v2014b script. BMI was specifically matched to account for previous findings that show lower BMI is associated with a greater risk of sustaining a concussion [35], and higher BMI is associated with reduced cognitive performance in athletes [33]. As the two concussions groups had the smallest number of participants for both sexes, this became the limiting factor for the size of the other matching groups. Case matching resulted in a total of 204 males, 68 per concussion groups, and 99 females, with 33 participants in each concussion group (see Additional file 1). A one-way analysis of variance (ANOVA) was used to examine overall differences between concussion groups on symptoms reported and scores obtained on the ImPACT neurocognitive battery composites. This was followed by Games-Howell post hoc analysis to account for disparity in variances across groups. To correct for multiple comparisons the MATLAB function *mafdr* was used to calculated the false discovery rate (FDR) for the p-values obtained by the Games-Howell post hoc analysis using the procedure introduced by Storey [36]. Dependent variables examined were Verbal Memory Composite, Visual Memory Composite, Visual Motor Composite, Reaction Time Composite, Impulse Control Composite, Total Symptom Score, and the 22 concussion-related symptom sub-measures in the PCSS. Significance for analyses was set a priori at *p* < 0.05, and resulted in a FDR < 0.05. Partial-eta squared values were calculated as measure of effect size, with 0.01 constituting a small effect, 0.06 a medium effect, and 0.14 a large effect [37].

## Results

### Males

After applying exclusion criteria and case matching of male subjects based on the number of previous concussions 204 participants remained, with 68 participants in each concussion group (0, 1, and 2 previous concussions). Demographics for the male groups are shown in Table 1.

**Table 1:** Group demographics for case matched males

Sport	0 Concussions (*n* = 68)	1 Concussion (*n* = 68)	2 Concussions (*n* = 68)
Football	37 (54.4%)	28 (41.2%)	38 (55.9%)
Basketball	10 (14.7%)	5 (7.4%)	4 (5.9%)
Baseball	5 (7.4%)	11 (16.2%)	8 (11.8%)
Cross-country	5 (7.4%)	3 (4.4%)	0 (0%)
Soccer	4 (5.9%)	5 (7.4%)	5 (7.4%)
Lacrosse	3 (4.4%)	5 (7.4%)	6 (8.8%)
Track and Field	2 (2.9%)	2 (2.9%)	0 (0%)
Wrestling	1 (1.5%)	2 (2.9%)	2 (2.9%)
Volleyball	1 (1.5%)	2 (2.9%)	1 (1.5%)
Swimming	0 (0%)	3 (4.4%)	2 (2.9%)
Golf	0 (0%)	2 (2.9%)	1 (1.5%)
Tennis	0 (0%)	0 (0%)	1 (1.5%)
Mean age (Std. Err.)	15.9 (0.14) yrs.	15.9 (0.14) yrs.	15.9 (0.14) yrs.
Mean BMI (Std. Err.)	23.14 (0.44)	23.12 (0.44)	23.14 (0.44)

Group comparisons of mean Total Symptom Score and the five ImPACT composite scores were conducted via a one-way ANOVA (Table 2). Results showed that a significant between-subjects main effect was not found for verbal memory, visual memory, visual motor, reaction time, or impulse control; however, a significant between-subjects main effect was found for total symptom score. Additionally, one-way ANOVA of the time since last concussion for the one concussion and two concussions groups showed no significant difference.

**Table 2:** One-way ANOVA results for ImPACT composite measures of case matched males stratified by number of previous concussions

Measure	0 Concussions	1 Concussion	2 Concussions	F[2,201]	*p*	Partial-eta squared
Verbal memory	82.65(1.17)	79.76 (1.21)	81.97 (1.31)	1.494	0.227	0.014
Visual memory	71.22 (1.49)	69.81 (1.78)	68.85 (1.71)	0.512	0.600	0.005
Visual motor speed	33.50 (0.86)	34.97 (0.84)	33.96 (0.92)	0.747	0.475	0.007
Reaction time	0.63 (0.01)	0.63 (0.01)	0.67 (0.02)	1.826	0.164	0.018
Impulse control	7.10 (0.63)	7.19 (0.63)	6.20 (0.51)	0.936	0.394	0.009
**Total symptom score**	**2.78 (0.55)**	**3.87 (0.63)**	**5.97 (.994)**	**4.666**	**0.010**	**0.044**
Mean time since last concussion (Std. Err.)	N/A	2.0 (1.7) yrs.	2.3 (2.4) yrs.	0.46^a^	0.502	0.005

Mean (Std. Err.). Bolded font indicates significant differences among concussion groups (*p* < 0.05)

^a^F[1,88]

**Fig. 1:**
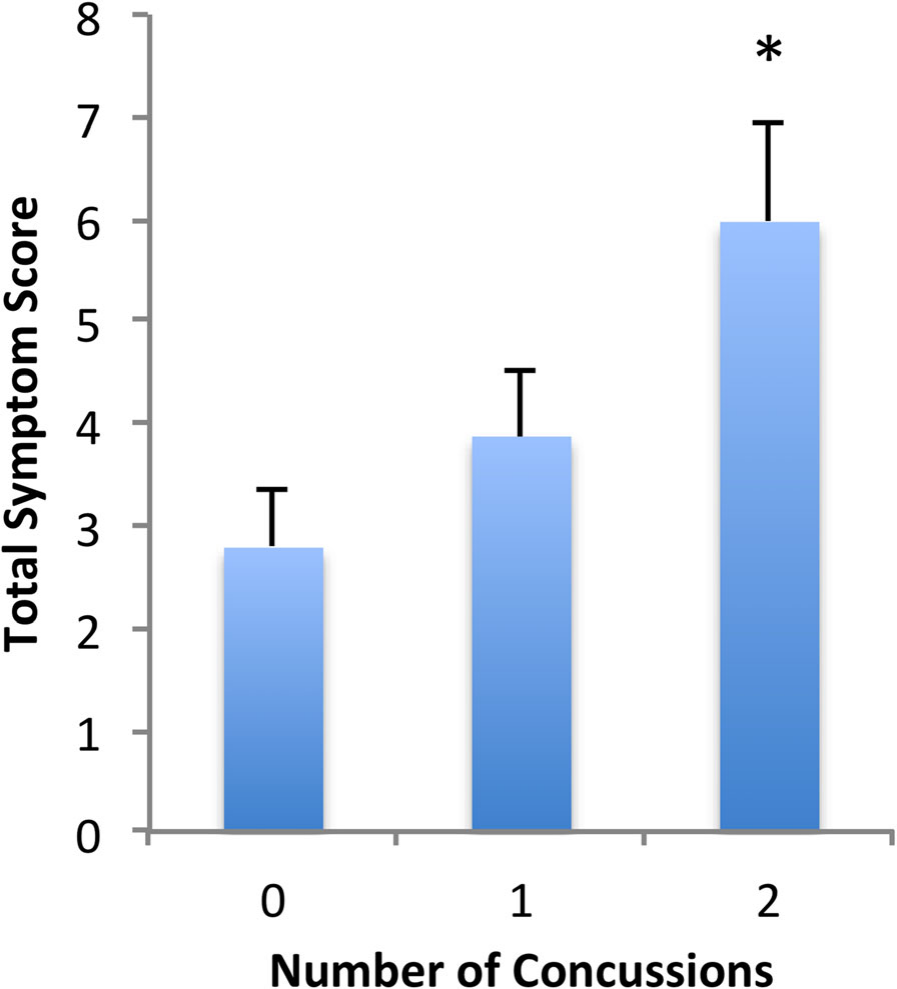
Mean and Standard Error of Total Symptom Score vs. Number of previous concussions for case matched males. As determined from Games-Howell post-hoc analysis, * indicates significant (*p* < 0.05) difference from the 0 concussions group

Games-Howell post-hoc of the total symptom score (Fig. 1) indicated there was a significant difference between those who had no previous concussion and two previous concussions, with the two previous concussion total symptoms scores being more than twice the zero concussion group’s total symptom scores. One previous concussion did not differ significantly from either groups.

**Fig. 2:**
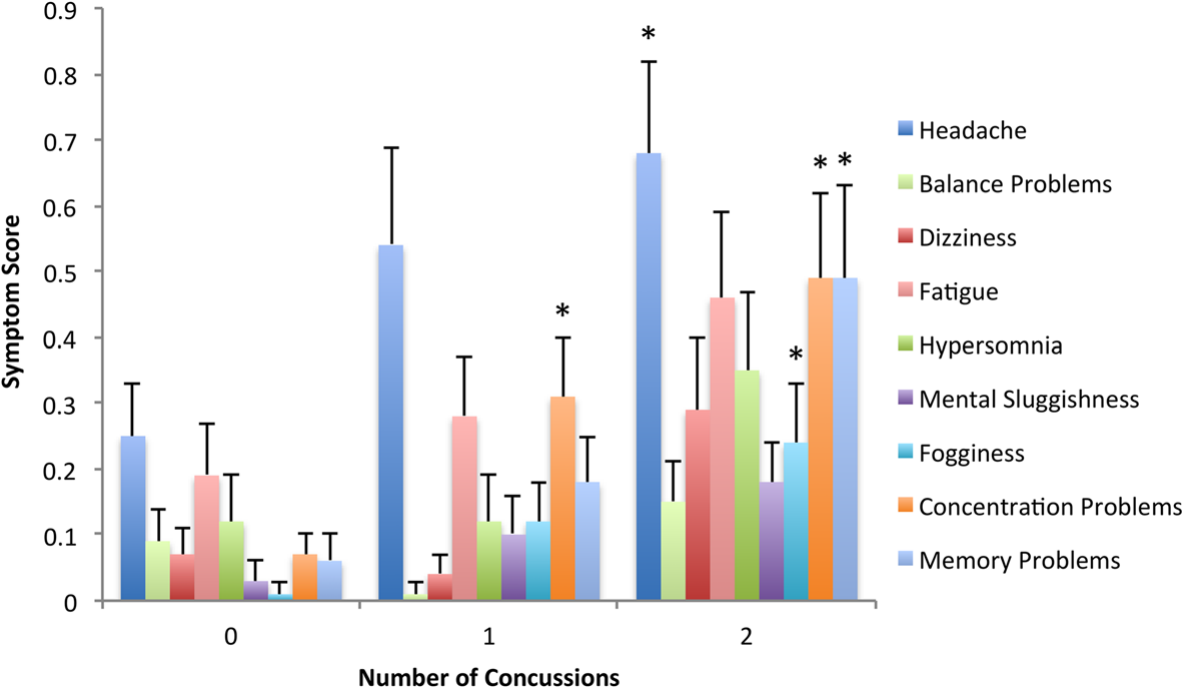
Mean and Standard Error of Post-Concussion Symptom Scale (PCSS) Measures vs. Number of Previous Concussions for case matched males. As determined from Games-Howell post-hoc analysis, * indicates significant (*p* < 0.05) difference from the 0 concussions group, and † indicates significant (*p* < 0.05) difference from both 0 concussions and 1 concussion groups. Only symptoms with *p* < 0.20 shown

As the composite Total Symptom Score was shown to differ across groups, further analysis of the 22 concussion-related symptom sub-measures in the PCSS was conducted via a one-way ANOVA (Table 3). Results showed that a significant between-subjects main effect was found for dizziness, fogginess, concentration problems, and memory problems. Games-Howell post hoc analysis of these groups (Fig. 2) indicated that increasing number of concussions increased the severity of reported symptoms.

**Table 3:** One-way ANOVA results for ImPACT Post-Concussion Symptom Scale (PCSS) measures of case matched males stratified by number of previous concussions

Measure	0 Concussions	1 Concussion	2 Concussions	F[2,201]	*p*	Partial-eta squared
Headache	0.25 (0.08)	0.54 (0.15)	0.68 (0.14)	2.875	0.059	0.028
Nausea	0.01 (0.02)	0.09 (0.06)	0.09 (0.05)	0.882	0.416	0.009
Vomiting	0.03 (0.02)	0.09 (0.04)	0.07 (0.04)	0.710	0.493	0.007
Balance problems	0.09 (0.05)	0.01 (0.02)	0.15 (0.06)	2.227	0.110	0.022
**Dizziness**	**0.07 (0.04)**	**0.04 (0.03)**	**0.29 (0.11)**	**3.666**	**0.027**	**0.035**
Fatigue	0.19 (0.08)	0.28 (0.09)	0.46 (0.13)	1.688	0.187	0.017
Initial insomnia	0.22 (0.09)	0.38 (0.12)	0.37 (0.11)	0.712	0.492	0.007
Hypersomnia	0.12 (0.07)	0.12 (0.07)	0.35 (0.12)	2.388	0.094	0.023
Hyposomnia	0.28 (0.11)	0.49 (0.14)	0.49 (0.14)	0.819	0.442	0.008
Drowsiness	0.07 (0.06)	0.15 (0.06)	0.21 (0.08)	0.965	0.383	0.010
Photophobia	0.26 (0.09)	0.24 (0.08)	0.21 (0.64)	0.131	0.878	0.001
Phonophobia	0.01 (0.02)	0.10 (0.05)	0.13 (0.06)	1.649	0.195	0.016
Irritability	0.12 (0.07)	0.16 (0.07)	0.13 (0.06)	0.123	0.884	0.001
Sadness	0.31 (0.10)	0.16 (0.07)	0.38 (0.10)	1.464	0.234	0.014
Nervousness	0.10 (0.07)	0.04 (0.03)	0.20 (0.07)	1.571	0.210	0.015
Emotionality	0.15 (0.08)	0.13 (0.07)	0.10 (0.05)	0.110	0.896	0.001
Numbness/Tingling	0.13 (0.07)	0.01 (0.02)	0.10 (0.05)	1.461	0.235	0.014
Mental sluggishness	0.03 (0.03)	0.10 (0.06)	0.18 (0.06)	2.047	0.132	0.020
**Fogginess**	**0.01 (0.02)**	**0.12 (0.06)**	**0.24 (0.09)**	**3.312**	**0.038**	**0.032**
**Concentration problems**	**0.07 (0.03)**	**0.31 (0.09)**	**0.49 (0.13)**	**5.4.855**	**0.009**	**0.046**
**Memory problems**	**0.06 (0.04)**	**0.18 (0.07)**	**0.49 (0.14)**	**5.887**	**0.003**	**0.055**
Visual problems	0.18 (0.07)	0.12 (0.07)	0.19 (0.07)	0.307	0.736	0.003

Mean (Std. Err.). Bolded font indicates significant differences among concussion groups (*p* < 0.05; FDR < 0.05)

### Females

After applying exclusion criteria and case matching of female subjects, 99 participants remained, with 33 participants in each concussion group (0, 1, and 2 or more previous concussions). Demographics for the female groups are shown in Table 4. Similar to the aforementioned analyses of males, group comparisons of mean Total Symptom Score and the five ImPACT composite scores were conducted via a one-way ANOVA (Table 5). Results showed no significant between-subjects main effect for verbal memory, visual memory, visual motor, reaction time, or impulse control, or total symptom score. One-way ANOVA of the time since last concussion for the one concussion and two concussions groups showed no significant group difference. Since no composite scores were significant, no further post-hoc analysis was performed.

**Table 4:** Group demographic table for case matched females

Sport	0 Concussions	1 Concussion	2 Concussions
	*N* = 33	*N* = 33	*N* = 33
Basketball	1 (3.0%)	2 (6.1%)	4 (12.1%)
Cheerleading	8 (24.2%)	7 (21.2%)	12 (36.4%)
Cross-country	1 (3.0%)	1 (3.0%)	2 (6.1%)
Football	2 (6.1%)	3 (9.1%)	1 (3.0%)
Lacrosse	1 (3.0%)	2 (6.1%)	1 (3.0%)
Other	1 (3.0%)	2 (6.1%)	1 (3.0%)
Soccer	4 (12.1%)	5 (15.2%)	6 (18.2%)
Softball	1 (3.0%)	6 (18.2%)	2 (6.1%)
Swimming	1 (3.0%)	1 (3.0%)	0 (0%)
Volleyball	12 (36.4%)	3 (9.1%)	4 (12.1%)
Water Polo	1 (3.0%)	1 (3.0%)	0 (0%)
Mean age (Std. Err.)	15.7 (0.2) yrs.	15.8 (0.2) yrs.	15.7 (0.2) yrs.
Mean BMI (Std. Err.)	21.4 (0.8)	21.2 (0.7)	21.4 (0.8)

**Table 5:** One-way ANOVA results for ImPACT composite measures of case matched females stratified by number of previous concussions

Measure	0 Concussions	1 Concussion	2 Concussions	F[2,98]	*p*	Partial-eta squared
Verbal memory	82.45(1.47)	83.85 (1.63)	83.06 (1.53)	0.205	0.815	0.004
Visual memory	70.36 (2.38)	68.30 (2.09)	72.97 (2.57)	0.988	0.376	0.020
Visual motor speed	35.86 (1.09)	37.03 (1.08)	38.55 (0.96)	1.671	0.193	0.034
Reaction time	0.615 (0.16)	0.613 (0.02)	0.60 (0.01)	0.199	0.820	0.005
Impulse control	5.61 (0.62)	5.00 (0.61)	5.73 (0.70)	0.366	0.695	0.008
Total symptom score	3.85 (1.20)	6.18 (1.46)	6.70 (1.64)	1.103	0.336	0.022
**Mean time since last concussion (Std. Err.)**	N/A	3.0 (3.9) yrs.	2.0 (1.4) yrs.	1.42^a^	0.2399	0.030

^a^F[1,46]

Mean (Std. Err). Bolded font indicates significant differences among concussion groups (*p* < 0.05)

## Discussion

In the present study, we assess the lingering effects of multiple past concussions on neurocognitive test performance and self-reported concussive symptoms. Starting from a large sample database of baseline neuropsychological profiles and post concussive symptom reports, we applied a stringent inclusion/exclusion criteria followed by case matching on age and BMI for each sex, separately. Overall, we find that male adolescent athletes’ baseline reports of symptoms associated with post concussive syndrome increase with the number of previous concussions. A similar trend was not found for adolescent female athletes. No significant change in neuropsychological measures was found regardless of sex.

These findings must be taken in the context of previous work in this area. Several past studies have shown varying significant associations between concussion, post-concussion symptoms, and neuropsychological performance in adolescent and young adult athletes [5, 9–12, 17]. These mixed results can often be attributed to small sample sizes leading to mixing of the sexes, lack of stringent inclusion/exclusion criteria, and absence of case matching sexes. The mixing of sexes is of particular concern [10, 15], as differences in BMI and structural build between the sexes may have bearing on concussion severity [32]. Seemingly in contrast to this, Brooks et al. [9] found that the number of previous concussions was significantly and positively correlated with the number of symptoms reported by a mixed sex population of adolescent athletes. While this agrees with our findings in males, it seems to suggest a disparity for females. However, it must be noted that while the Brooks et al.’s study [9] was of mixed sex, their cohort was 83.8% male, and therefore significantly biased against females, and not likely capturing the trends in their response to multiple concussion. Separate analysis of the effects of multiple concussions on females is required.

Lack of stringent inclusion/exclusion criteria and absence of case matching are also highly problematic. Iverson et al. [8] used a high school and collegiate male athlete population to examine the effects of one or two previous concussions on participants’ neurocognitive functioning, finding there were no measurable effects. As our stringent case matched study results show, the two previous concussions groups reports significantly higher negative symptom effects, suggesting the unaffected symptom reports found in athletes by Iverson et al. [8] may be due to a lack of exclusionary criteria and/or lack of case matching beyond accounting for sex. Furthermore, it must also be noted that compared to our study, the participant group in the Iverson et al. [8] study had a slightly higher age range (13–22 years, mean 17.7 years), a different distribution of sports played by participants (although football was predominant in both studies), and no information on the time since the last previous concussion. All of these differing factors could affect the severity of concussion symptoms identified.

A similar study by the same group [11] compared male athletes aged 17–22 that had a history of three or more concussions to similarly aged athletes with no history of concussion. They found the previously concussed athletes performed significantly worse on a verbal memory task, although no significant difference in total symptoms were identified. Coupled with our results showing no change in verbal memory with two previous concussions, these findings suggest that at least three previous concussions are required before negative effects of concussions are evident in verbal memory. However, these results are also in contrast to our findings that two previous concussions may contribute to persistent symptom effects, as three previous concussions did not produce significant changes in symptoms. While this study case matched on sex; age; education; self-reported ADHD; school; sport; and, when possible, playing position and self-reported academic problems, it did not exclude participants self-reporting ADD, ADHD, academic, or learning problems; as has previously been recommended [10, 15, 17–21], leaving this as a possible explanation for the observed difference between our studies. However, as mentioned above, differences in age, time from last concussion, and sports the participants played between the studies could be factors that affect the severity of concussion symptoms reported. Regardless of the number of concussions needed before the onset of identifiable negative consequences, there is a growing number of research studies indicating that persistent effects of concussions can occur much earlier in the life-span than previously thought [5, 8, 10, 38]. Collectively, these findings support the notion that concussion in adolescent and college athletes may lead to long-term complications in this relatively young and healthy population.

The findings from the present study suggest male athletes reporting multiple past brain injuries suffer from greater daily discomfort than their non-concussed peers. The increased endorsement of dizziness, fogginess, concentration problems and memory problems may indicate chronic symptoms of head injury sequelae in the male sample. However, this pattern is not evident in the female sample, which is contradictive to research reporting more severe symptomology and longer recovery in females when compared to males [31]. It is not clear why a similar trend was not observed in females, although several possible explanations exist. One explanation may be that male athletes are known to underreport symptoms and are less likely to be diagnosed with a concussion unless a relatively severe or higher number of symptoms are present. A second explanation may be the limitation of the ImPACT only examinings subjective accounts of previous concussions and symptoms. As a result, males endorsing more symptoms may also be more likely to connect their experiences to a higher number of past concussions than their peers. A third explanation may be the difference in sports played by male and female athletes, which may affect the severity of concussions received. For example, males being more likely to engage in sports associated with increased violent and aggressive acts (e.g., football and wrestling). Lastly, previous research has suggested that the female sex hormones, estrogen and progesterone, are neuroprotective and may aid in their recovery post-concussion [39, 40].

The absence of differences amongst cognitive symptoms is not unprecedented, as studies have not consolidated the potential consequences of multiple head injury sequeale [6]. Each individual has constellation biological, psychological, and social factors that could either exacerbate or mitigate the onset of serious head-injury consequences [41]. However, it should be noted that the ImPACT test is a screening tool that is designed to assess cognitive symptoms that aid in diagnosis of concussion. As it is designed to measure cognitive symptoms in acute head injury, it may not have the necessary sensitivity and specificity to measure chronic changes in cognition [19].

The use of a strict inclusion and exclusion criteria combined with a case matched design represents perhaps the most significant strength of this study. While some concussion research has employed large community-based samples [42], the majority of the published research on concussion and mTBI have used samples of relative small sizes and homogeneous groups. The use of baseline data, rather than post-injury data, in the empirical investigation of concussion-related concepts is also relatively unique in mTBI literature. In both of these regards, as well as in the statistical analyses employed herein, the present research study has sought to add a novel approach to concussion-related research. This study also demonstrated evidence of confounding factors (e.g., age, sex, BMI, psychosocial factors) possibly impacting neurocognitive performance following self-reported concussions, causing variable results to be found amongst the literature. When stringent inclusion/exclusion criteria and a case controlled design was utilized, no significant differences were found amongst neurocognitive performance. Our results tend to contradict a significant portion of the current literature, providing further evidence of the role confounding factors may be playing and the importance of strict inclusion and exclusion criteria when examining the effects of concussion. Consequently, we recommend use of caution when interpreting research that utilizes a relatively lenient inclusion and exclusion criteria (e.g., not screening for learning disorder diagnoses and ADHD) as well as the analysis of results that neglected to control for differences among gender, BMI, age, etc.

### Limitations

Despite this, there are limitations to the present study that need to be acknowledged. First, the data did not wholly capture concussion recency effects. While the average time since last concussion was accounted for, this data was not complete, and the risk for persistent post-concussive symptoms and neurocognitive impairment following multiple concussions may depend on how close together the events occurred. Second, due to limitations of information collected by the ImPACT, it is unclear whether some participants were currently experiencing concussive symptoms during baseline testing, causing this to be a possible confounding factor. Third, the present study is also challenged by the reliance on retrospective self-report and potential limitations of the ImPACT. This limitation is of particular concern as significant differences were only found on subjective measures (i.e., self-reported number of concussions and concussive symptoms), with no significant differences found on the ImPACT’s objective measures (i.e., neurocognitive measures). This finding is further scrutinized due to the conflicting nature of symptoms reported (e.g., complaints of dizziness, but not balance or visual problems; complaints of concentration and memory problems, but not mental sluggishness). It is also possible that individuals who fixate on symptoms are more likely to report previous concussions and/or receive concussion diagnoses by a clinician, whereas those who downplay or ignore their symptoms are less likely to receive a diagnosis. However, a significant body of research has shown athletes generally underreport concussive symptoms [43–45]. Until objective concussion diagnostic measures are found, researchers must be vigilant of possible extraneous psychosocial factors.

## Conclusion

Ultimately, due to the self-report method by which information on previous concussions was obtained, this study design does not allow for an inference of causation between concussion history and persistent neuropsychological impairment or post-concussive symptoms. However, our results did provide evidence of persisting negative subjective effects that correlate with the number of self-reported previous concussions, suggesting a causative relation. Therefore, future research in this area is warranted.

## Additional file

Additional file 1: Supplementary Information. Data Description: De-identified case matched data of ImPACT measures used to generate all results of this study. (XLS 108 kb)
